# Enzymatic production of 4-*O*-methyl d-glucaric acid from hardwood xylan

**DOI:** 10.1186/s13068-020-01691-2

**Published:** 2020-03-13

**Authors:** Thu V. Vuong, Emma R. Master

**Affiliations:** 1 Department of Chemical Engineering and Applied Chemistry, University of Toronto, Toronto, ON Canada; 2 Department of Bioproducts and Biosystems, Aalto University, Aalto, Kemistintie 1, 00076 Espoo, Finland

**Keywords:** 4-*O*-Methyl d-glucaric acid, GH115, AA7, Xylan, Hemicellulose, Biorefinery

## Abstract

**Background:**

Dicarboxylic acids offer several applications in detergent builder and biopolymer fields. One of these acids, 4-*O*-methyl d-glucaric acid, could potentially be produced from glucuronoxylans, which are a comparatively underused fraction of wood and agricultural biorefineries.

**Results:**

Accordingly, an enzymatic pathway was developed that combines AxyAgu115A, a GH115 α-glucuronidase from *Amphibacillus xylanus*, and GOOX, an AA7 gluco-oligosaccharide oxidase from *Sarocladium strictum*, to produce this bio-based chemical from glucuronoxylan. AxyAgu115A was able to release almost all 4-*O*-methyl d-glucuronic acid from glucuronoxylan while a GOOX variant, GOOX-Y300A, could convert 4-*O*-methyl d-glucuronic acid to the corresponding glucaric acid at a yield of 62%. Both enzymes worked effectively at alkaline conditions that increase xylan solubility. Given the sensitivity of AxyAgu115A to hydrogen peroxide and optimal performance of GOOX-Y300A at substrate concentrations above 20 mM, the two-step enzyme pathway was demonstrated as a sequential, one-pot reaction. Additionally, the resulting xylan was easily recovered from the one-pot reaction, and it was enzymatically hydrolysable.

**Conclusions:**

The pathway in this study requires only two enzymes while avoiding a supplementation of costly cofactors. This cell-free approach provides a new strategy to make use of the underutilized hemicellulose stream from wood and agricultural biorefineries.

## Background

Glucuronoxylans are a major form of hemicellulose present in wood and agricultural fiber. For example, between 15 and 30% of hardwood dry mass is acetylated–glucuronoxylan, whereas up to 10% of coniferous softwood dry mass is arabino-glucuronoxylan, and up to 30% of cereal dry mass is glucurono-arabinoxylan [1]. In these glucuronoxylans, the 4-*O*-methyl d-glucuronic acid (MeGlcA) or d-glucuronic acid (GlcA) substituents are linked to the β-(1→4)-linked xylose backbone through an α-(1→2)-linkage. The ratio of (Me)GlcA to xylose (Xyl*p*) depends on the xylan source, and is mainly reported as being between 1:10 [2] and 1:15 [3], but also as high as 1:3 [1], in glucuronoxylan from hardwoods. The varied chemistry of xylans has posed a challenge to their broader use, and xylans remain a comparatively underused fraction of wood and agricultural fiber. Enzymatic release of MeGlcA present in glucuronoxylans, however, could be performed as the first step to methyl glucaric acid production while facilitating the recovery of a simplified xylan stream suitable for conversion to xylitol or high-value products.

Glucaric acid was listed by the US Department of Energy in 2004 as one of the top 12 bio-based chemicals [4]. This dicarboxylic acid could replace phosphoric acid as a builder component in detergents for calcium and magnesium sequestering [5]. It is also a potential building block for a number of biopolymers including new nylons and hyperbranched polyesters [6]. Methyl groups of monomers contributed to the molecular architecture and subsequent properties of their derived biopolymers [7]. Therefore, the methylated form of glucaric acid from glucuronoxylan could bring additional functional properties to the chemical, including higher compatibility with surfactants in detergents and hydrophobic biopolymers.

Presently, glucaric acid is commercially synthesized as glucarate by the non-selective nitric acid oxidation of glucose with a yield of ca. 40% [8]. This conventional approach as well as recent heterogeneous, metal catalyst methods [9, 10] suffers from low selectivity, increasing the cost for downstream separation of glucaric acid from other organic acid by-products [11]. The absence of green technologies for glucaric acid production is one of the reasons for its exclusion from the revised list of new top chemical opportunities from biorefineries [12]. Accordingly, considerable investment has been focused on engineering microorganisms such as *E. coli* [13] and *Saccharomyces cerevisiae* [14] to transform glucose into glucaric acid. However, even when a co-substrate, *myo*-inositol was added, the maximum yield was only 56% [14], which remains inefficient for industrial scale production of glucaric acid. Furthermore, fermentation approaches can complicate product recovery, due to the presence of medium components and other metabolites/intermediates. A cell-free approach to produce 4-*O*-methyl d-glucaric acid (or methyl glucaric acid, for short) from glucuronoxylan was reported [15], where three enzymes including an endo-xylanase (EC 3.2.1.8), α-glucuronidase (EC 3.2.1.139), and uronate dehydrogenase (EC 1.1.1.203) were used in a cocktail or co-localized on a scaffold. The xylanase cleaved glucuronoxylan to various xylo-oligosaccharides, of which some contained MeGlcA. The α-glucuronidase then removed MeGlcA residues that were attached to the non-reducing end of short xylo-oligosaccharides. The released MeGlcA was finally converted to methyl glucaric acid by the dehydrogenase [15]. Notably, this approach requires a continuous supply of an exogenous cofactor (NAD^+^) and the separation of methyl glucaric acid from other soluble xylo-oligosaccharides. To avoid the need for supplementation with expensive and labile NAD^+^, other in vitro pathways were proposed that utilize seven enzymes, including uronate dehydrogenase and NADH oxidase, for the production of glucaric acid from sucrose [16, 17]. However, these multi-enzyme cascades require an initial amount of NAD^+^ and a continuous supply of inorganic phosphate. It was also noted that uronate dehydrogenase used in such pathways can release glucaric acid 1,4-lactone rather than the open-ring form [18].

**Fig. 1:**
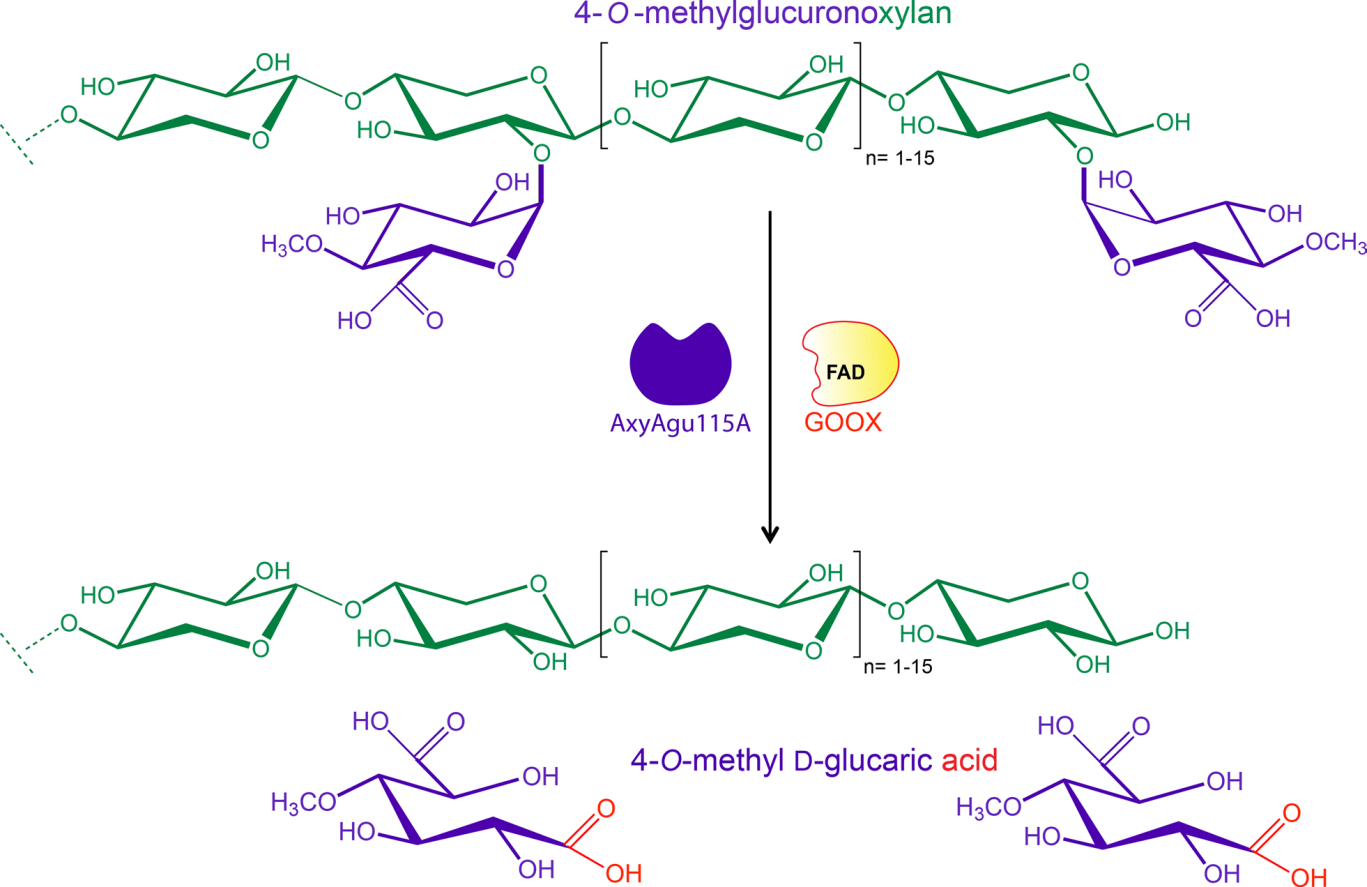
The proposed two-enzyme pathway for 4-*O*-methyl glucaric acid production from glucuronoxylan

A GH115 α-glucuronidase from *Amphibacillus xylanus* (AxyAgu115A), which hydrolyzes the α-(1→2)-linkage connecting MeGlcA to internal and terminal substituted regions of xylans [19], has been previously characterized by our group. This enzyme displays the highest relative activity in alkaline conditions among all other characterized GH115 α-glucuronidases with known pH profiles [19]. Meanwhile, we have also characterized a gluco-oligosaccharide oxidase from *Sarocladium strictum* (GOOX) [20], which is a member of Auxiliary Activities family 7 (AA7, http://www.cazy.org). GOOX oxidizes a number of monosaccharides and oligosaccharides, including those derived from hemicelluloses [20]. Seventeen GOOX variants were created [20–22], of which, the catalytic efficiency of GOOX-Y300A on glucose is nearly four times higher than that of the wild-type [20]. Like AxyAgu115A, GOOX works effectively at alkaline conditions with the optimum pH of 10 [23]. In this study, AxyAgu115A and GOOX-Y300A were combined into a sequential one-pot reaction to produce 4-*O*-methyl d-glucaric acid from glucuronoxylan, without cofactor supplementation (Fig. 1). In addition to this methyl glucaric acid, a simplified, hydrolysable xylan stream was also recovered.

## Results and discussion

### Release of 4-*O*-methyl d-glucuronic acid by AxyAgu115A

AxyAgu115A was produced with greater than 95% purity (Additional file 1: Figure S1). Glucuronoxylan is more soluble in alkaline conditions, allowing 6% glucuronoxylan to be incubated with AxyAgu115A with proper mixing. AxyAgu115A released only MeGlcA from glucuronoxylan, as analyzed by HPAEC-PAD (Additional file 1: Figure S2). Most of MeGlcA was released by AxyAgu115A during 4 h of incubation (> 98%), and no additional release of MeGlcA was seen after 16 h. The half-life of AxyAgu115A at 40 °C was 24 h [19], thus the enzyme remained active during the 16-h hydrolysis. Consistent with earlier findings [3], no trace of GlcA release (the unmethylated form) was detected, even though the sensitivity of HPAEC-PAD for GlcA detection is approximately three times higher than for MeGlcA (Additional file 1: Figure S3). The release of MeGlcA was also confirmed by nanospray ionization ion-trap mass spectrometry (NSI-MS). A mass scan from 100 to 1000 m/z showed that MeGlcA and its dimer are the two major peaks in the spectrum (Additional file 1: Figure S4). The simulated spectrum of MeGlcA was also matched well to the acquired spectrum (Additional file 1: Figure S5).

When the glucuronoxylan loading was greater than 6%, the reaction became viscous, preventing mixing; therefore, the concentration of glucuronoxylan was set at 6%. At this substrate loading, the concentration of MeGlcA released after 4 h (in 25-mL reactions) was 21.6 ± 1.2 mM, calculated based on the MeGlcA standard curve (Additional file 1: Figure S6). The theoretical maximum molar concentration of MeGlcA, based on a previous analysis of glucuronoxylan composition [3], was 24.4 mM; therefore, AxyAgu115A was able to release almost all of MeGlcA present in glucuronoxylan. This finding was supported by methanolysis, where the total concentration of MeGlcA released from glucuronoxylan was measured at approximately 15.6 mM. The nearly 20% lower concentration of MeGlcA released by methanolysis compared to the enzymatic treatment can be explained by partial MeGlcA degradation at high temperature (100 °C) and acid conditions (2 M HCl) required for methanolysis. A similar percentage of MeGlcA degradation by methanolysis was also previously reported [24].

The pKa of MeGlcA is 3.0, as predicted by ACD/Labs 2.0 v5 (http://www.ilab.acdlabs.com), so at alkaline conditions, MeGlcA is negatively charged. Therefore, anion-exchange chromatography was used to purify MeGlcA released by AxyAgu115A, which confirmed that the acidic sugar eluted from Dowex resin at ammonium acetate concentrations above 0.5 M (Additional file 1: Figure S7).

### Oxidation of 4-*O*-methyl d-glucuronic acid by GOOX variants

**Fig. 2:**
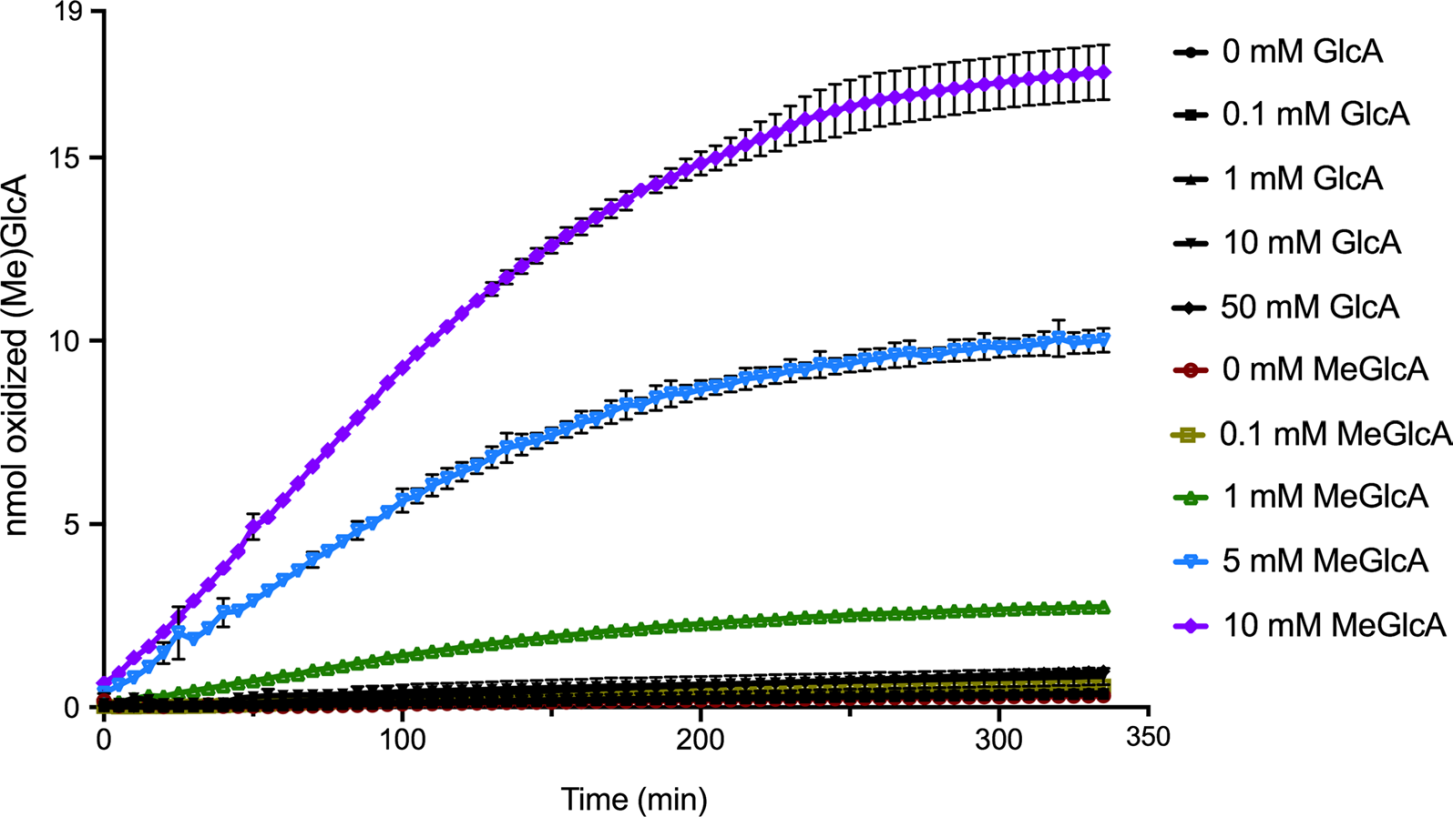
Substrate preference of GOOX-Y300A. The enzyme (16 nM) was incubated with different concentrations of MeGlcA and GlcA, up to 50 mM in 0.3 M Tris buffer pH 8 at 40 °C, and the amount of oxidized products was determined using a colorimetric assay [23]

**Fig. 3:**
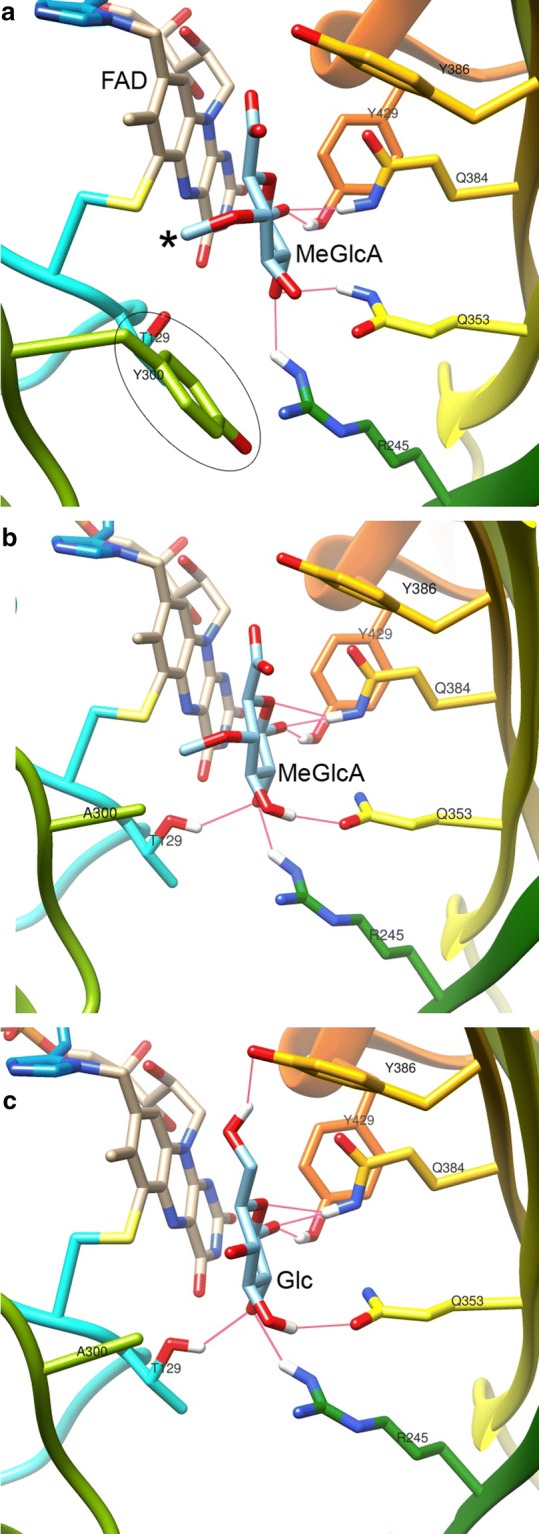
Docking of MeGlcA and glucose to GOOX variants. **a** MeGlcA formed four hydrogen bonds (pink line) in the wild-type enzyme, the methyl group (starred) pointed toward the Y300 residue (circled). **b** The replacement of Y300 with alanine allowed MeGlcA to form six hydrogen bonds. **c** Glucose (Glc) formed seven hydrogen bonds with GOOX-Y300A

A preliminary screening of 17 GOOX variants [20, 22] on GlcA and MeGlcA found that the methylated form of d-glucuronic acid was the preferred substrate (Additional file 1: Figure S8); notably, the commercial glucose oxidase did not show any activity on either GlcA or MeGlcA (Additional file 1: Figure S8). One of the GOOX variants, Y300A [20] showed higher specific activity on MeGlcA than other GOOX variants; the Y300A variant also preferred oxidation of MeGlcA over GlcA (Fig. 2). Docking analyses of MeGlcA to the wild-type and Y300A variant showed that the methyl group pointed toward Tyr300, and the replacement of tyrosine with alanine increased hydrogen-bonding to MeGlcA from four to six bonds (Fig. 3). Notably, seven hydrogen bonds are predicted between glucose and the Y300A variant, and performance of the Y300A variant on glucose is higher than that on MeGlcA (see below). This GOOX variant, hereafter GOOX-Y300A, was used for this study (Additional file 1: Figure S1).

**Fig. 4:**
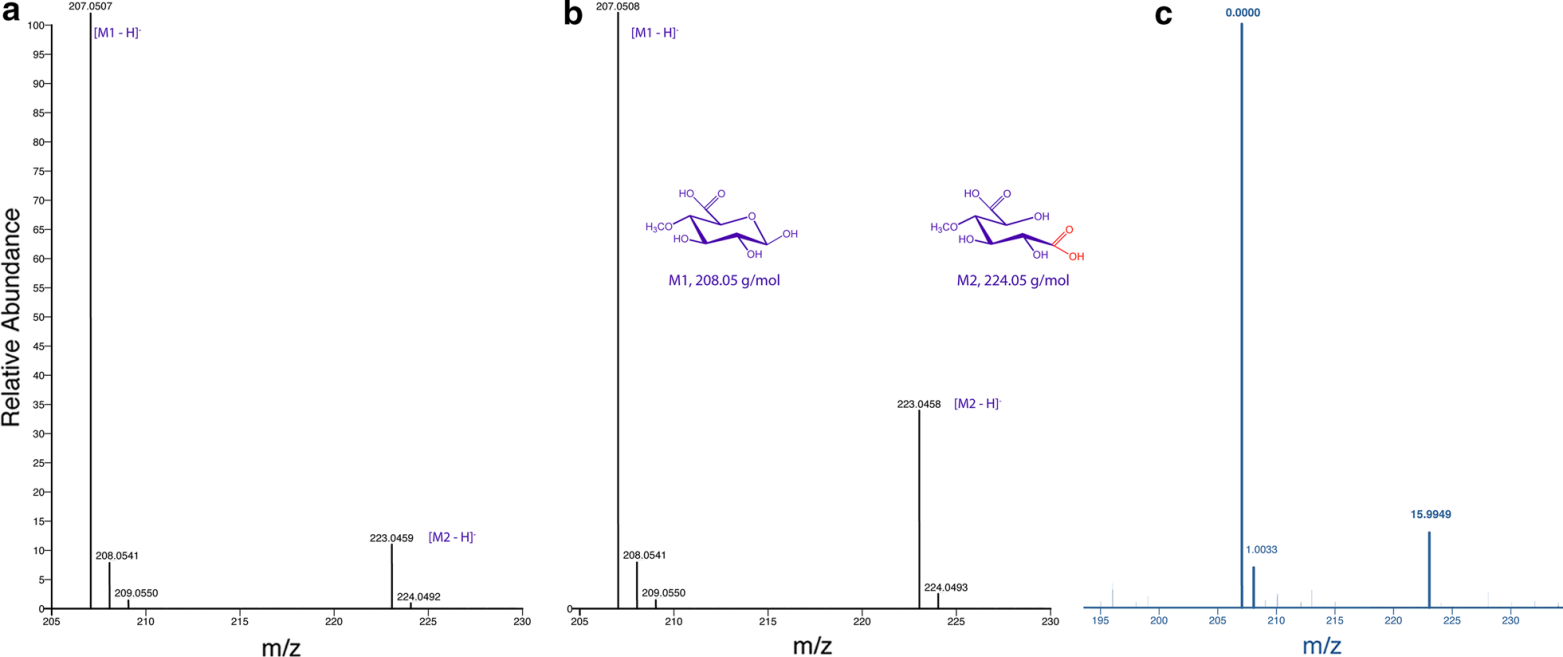
NSI-MS spectra for the formation of methyl glucaric acid (224.05 g/mol) by GOOX-Y300A. Mass spectra of GOOX-Y300A activity on 1 mM and 10 mM MeGlcA (**a**, **b**, correspondingly); notably, MeGlcA and methyl glucaric acid displayed different ionization potentials. The corrected mass spectrum to confirm the addition of one oxygen (15.9949 m/z) (**c**)

The formation of methyl glucaric acid by GOOX-Y300A was confirmed by NSI-MS (Fig. 4c), where methyl glucaric acid was seen at 223.05 m/z, confirming the oxidation (addition of 15.99 m/z) of MeGlcA (207.05 m/z). There was a dose response for the production of methyl glucaric acid, as the relative abundance of methyl glucaric acid increased when the substrate concentration was raised from 1 mM to 10 mM (Fig. 4a, b). Consistent with this observation, kinetic analysis of GOOX-Y300A on MeGlcA revealed a *K*_m_ of 21 ± 2 mM and *k*_cat_ of 0.91 ± 0.06 min^−1^. This *K*_m_ value is in the range of MeGlcA concentration that was fully released from 6% glucuronoxylan, suggesting that GOOX-Y300A should be added to the reaction after the complete action of AxyAgu115A. However, the *K*_m_ of GOOX-Y300A on MeGlcA was higher than that on glucose (8.1 mM) while its *k*_cat_ was nearly three orders of magnitude lower [20], highlighting the merits of further engineering GOOX-Y300A to increase catalytic performance on MeGlcA.

The conversion yield from MeGlcA to methyl glucaric acid by GOOX-Y300A was 62%; given methyl glucaric acid was not commercially available, yields were based on MeGlcA consumption quantified by HPAEC-PAD and LC–MS. This yield is 55% higher than the nitric acid oxidation of glucose to glucaric acid [8]. The conversion yield was not reported by Lee et al. [15], who proposed the in vitro pathway with three enzymes including endo-xylanase, α-glucuronidase, and uronate dehydrogenase to produce (methyl)glucaric acid from birchwood xylan. The authors reported a maximum production of 0.7 mM (methyl)glucaric acid from 1% birchwood xylan after more than 2 h, as quantified by NADH absorbance at 340 nm [15]. Provided that a similar xylan source was used, this would be equivalent to a conversion yield of ~ 20%. Using the same three-enzyme pathway, but with a new thermostable uronate dehydrogenase, Li et al. increased (methyl)glucaric acid yield from beechwood xylan to more than 80% [25]. Notably, both previously reported methods require a continuous supply of NAD^+^, motivating Su et al. to establish a seven-enzyme pathway to incorporate NAD^+^ regeneration [16]. Although the seven-enzyme pathway reached a conversion yield of up 75  % glucaric acid from sucrose in 72 h, the total enzyme loading was more than 2 mg/mL, instead of 0.01 mg/mL as in two-step pathway reported herein.

Methyl glucaric acid is also chemically produced from MeGlcA using Ca(OH)_2_ and NaOH; however, the highest yield was only 24%, and the final reaction solution contained eight other dicarboxylic acids [26]. Several approaches that use heterogeneous metal catalysts including Pt/C, Pt/Au, Au/C or AuBi/C or Pt_1_Cu_3_/TiO_2_ [9, 10] could gain a complete conversion of glucose. Unfortunately, the full selectivity of glucose to glucaric acid is not achievable, requiring a separation of glucaric acid from other oxidized products.

### Impacts of hydrogen peroxide

When GOOX-Y300A oxidizes carbohydrates, it utilizes molecular oxygen as a co-substrate, producing hydrogen peroxide. Therefore, the effects of H_2_O_2_ on MeGlcA and enzymatic stability must be considered. No loss of MeGlcA was observed at 100 mM H_2_O_2_ (Additional file 1: Figure S9A). Under our conditions, even at the highest glucuronoxylan loading, only about 20 mM H_2_O_2_ could be present as it depends on the released concentration of MeGlcA. Even at 200 mM H_2_O_2_, GOOX-Y300A retains more than 50% of its activity on glucose and 100% of its activity on cellobiose [27]. Furthermore, it was reported that H_2_O_2_ is less stable in alkaline conditions, particularly at elevated temperatures (40 °C) [28]. In contrast, AxyAgu115A activity was inactivated when the concentration of H_2_O_2_ was greater than 1 mM, and approximately half of AxyAgu115A activity was lost in the presence of 10 mM H_2_O_2_ (Additional file 1: Figure S9B).

### Sequential one-pot reaction for methyl glucaric acid production

**Fig. 5:**
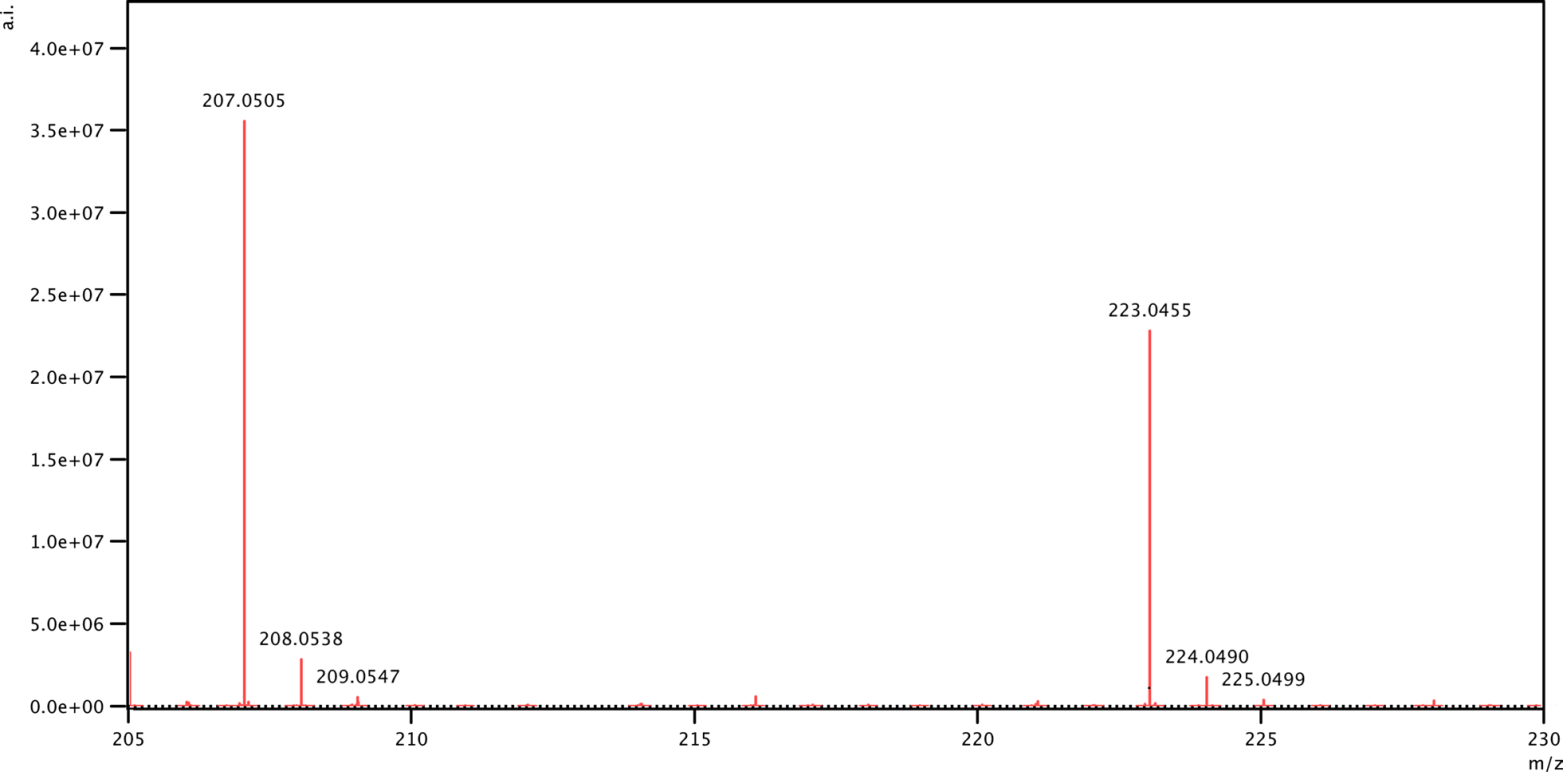
LC–MS of glucuronoxylan after a one-pot sequential treatment with AxyAgu115 and GOOX-Y300A. Both MeGlcA (207.05 m/z) and methyl glucaric acid (223.05 m/z) were detected in the negative mode; the absolute intensity (a.i.) was shown

Both AxyAgu115A and GOOX-Y300A act at high pH values and display a similar temperature profile [19, 23], confirming the compatibility of these enzymes. Conducting reactions at alkaline conditions offers several advantages, including a higher glucuronoxylan loading (6% in this study, compared to 1% reported in Lee et al. [15]) and a lower presence of lactone forms of glucaric acid [29], which could hinder product recovery. Because the *K*_m_ of GOOX-Y300A was at mM levels, and AxyAgu115A was partially inactivated by H_2_O_2_, a one-pot sequential reaction was performed where GOOX-Y300A was added to the reaction after pre-hydrolysis of glucuronoxylan by AxyAgu115A for 4 h. Following 16 h of incubation with GOOX-Y300A, methyl glucaric acid yields were similar to those achieved using the two-pot sequential system described above (i.e., 60% yield as confirmed by LC–MS; Fig. 5).

**Fig. 6:**
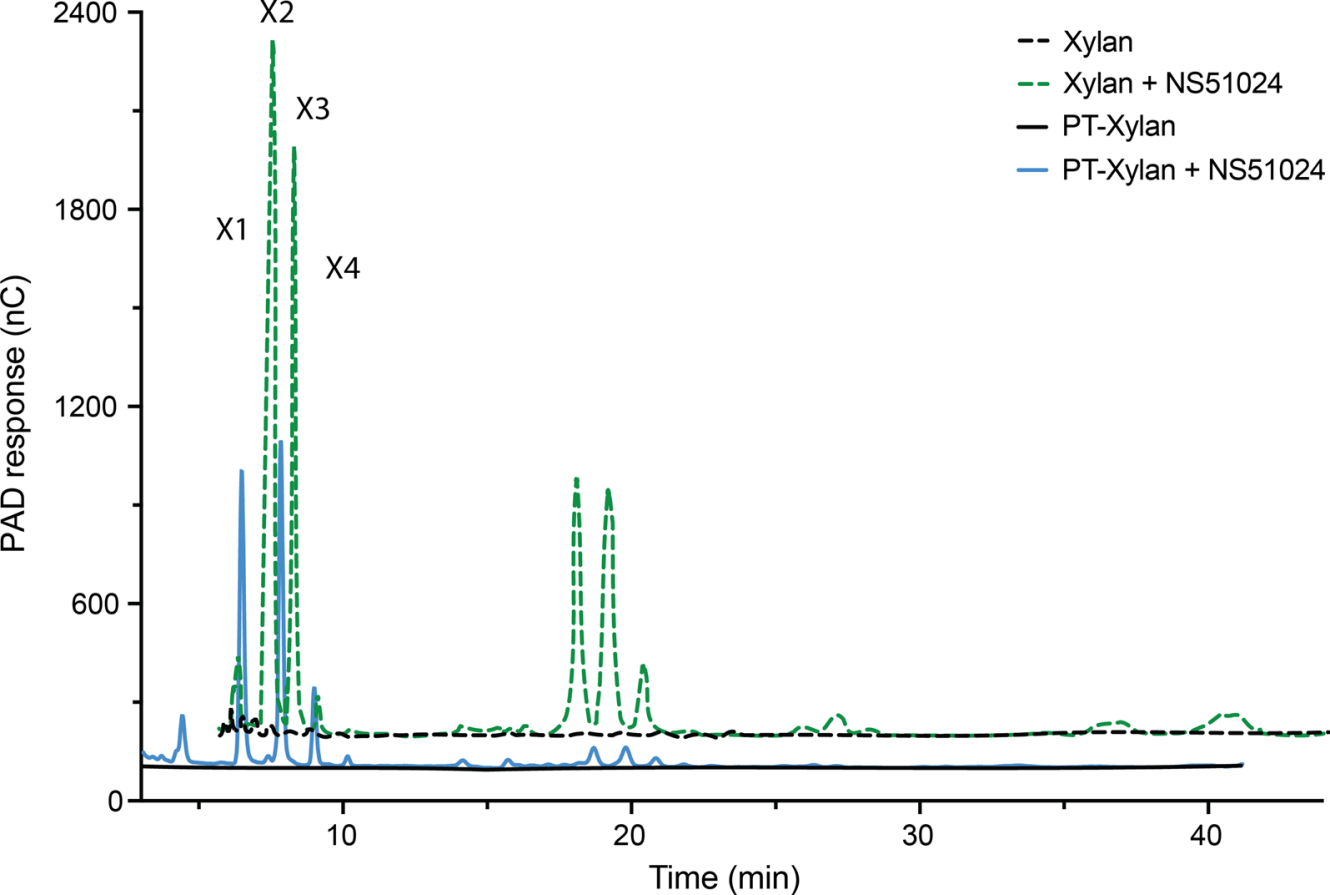
HPAEC-PAD analysis for xylanase digestion of xylan (dash line), as well as AxyAgu115A and GOOX-Y300A-pretreated xylan (solid line). Xylan samples were treated using Novozymes NS51024; X1–X4 are xylose, xylobiose, xylotriose, and xylotetraose correspondingly. Peaks eluting after 15 min correspond to larger and/or charged oligosaccharides

The xylan after AxyAgu115A and GOOX-Y300A treatments formed a hydrogel-like material (Additional file 1: Figure S10), which was separated from the reaction by quick centrifugation (1 min at 10,000×*g*). This effect on rheological properties was previously correlated to xylan debranching [30]. After washing with MQ water to remove any remaining soluble products, the resulting xylan was still hydrolysable by Novozymes xylanase NS51024 (GH11 xylanase from *Thermomyces lanuginosus*) (Fig. 6). HPAEC-PAD analyses revealed lower amounts of small oligosaccharides, and less abundance of large/charged oligosaccharide fractions in the resulting xylan (Fig. 6), which is in agreement with its lower reducing-end sugar yield (40%), compared with the starting glucuronoxylan (Additional file 1: Figure S11). This already washed and neutralized xylan could decrease downstream separation costs in applications that require high purity of xylan, for instance in xylitol production.

## Conclusion

We report an enzymatic pathway for the production of methyl glucaric acid from glucuronoxylan using two enzymes, AxyAgu115A and GOOX-Y300A. AxyAgu115A released most of MeGlcA directly from glucuronoxylan, as confirmed by methanolysis, whereas GOOX-Y300A converted MeGlcA to methyl glucaric acid at a yield of 62%. Both enzymes worked at alkaline conditions, which permitted reactions using 6% glucuronoxylan. Because the *K*_m_ value of GOOX-Y300A on MeGlcA was 21 ± 2 mM and AxyAgu115A showed evidence of inhibition above 1 mM H_2_O_2_, these enzymes are suitable for a sequential one-pot reaction where a similar conversion yield was achieved. The xylan co-product can be separated by centrifugation and readily hydrolyzed by a commercial xylanase to xylose and xylo-oligosaccharides, which might be suitable for xylitol or higher value applications.

## Materials and methods

### Materials

4-*O*-Methyl glucuronoxylan from beechwood, also known as glucuronoxylan (cat. no. M5144), was purchased from Sigma, USA. 4-*O*-methyl d-glucuronic acid (MeGlcA, purity > 95%, by 1H-NMR, cat. no. MG244) was purchased from Synthose Inc., Canada while d-glucuronic acid (GlcA, not methylated, purity > 98% by GC, cat.no. G5269) was purchased from Sigma, USA. Commercial enzymes used were catalase (cat. no. C40 from Sigma, USA, ≥ 10,000 units/mg protein), glucose oxidase (cat. no. G2133 from Sigma, USA), and xylanase (cat. no. NS51024 from Novozymes, Denmark).

### Protein production

AxyAgu115A and GOOX-Y300A were produced based on the previous publications [19, 22]. Briefly, for AxyAgu115A purification, *E. coli* BL21(λDE3) CodonPlus was grown at 37 °C in Luria–Bertani medium containing 500 mM sorbitol, 2.5 mM glycine betaine, 34 μg/mL chloramphenicol and 100 μg/mL ampicillin. Cells were induced by 0.5 mM IPTG at 15 °C for 16 h. Cells were then sonicated in a binding buffer (300 mM NaCl, 50 mM HEPES pH 7.0, 5% glycerol, and 5 mM imidazole). After centrifugation, the supernatant was incubated with Ni–NTA resin for 2 h at 4 °C, and the protein was eluted with an elution buffer (300 mM NaCl, 50 mM HEPES pH 7.0, 5% v/v glycerol, and 250 mM imidazole). The protein was further purified using a Bio-Gel P10 column. For GOOX-Y300A purification, the corresponding *Pichia pastoris* was grown in BMGY medium, and then induced in BMMY medium with 0.5% methanol added daily. The secreted GOOX-Y300A protein was harvested using Ni–NTA resin as described for AxyAgu115A. The concentration and purity of these recombinant proteins were determined by gel densitometry using a bovine serum albumin (Thermo Fisher Scientific, USA) as the standard. Other GOOX variants were produced in the previous work [20–22].

### Enzymatic hydrolysis and oxidation

Glucuronoxylan (6%) was incubated with AxyAgu115A (10 μg/mL, 91 nM) and GOOX-Y300A (10 μg/mL, 179 nM) in 100 mM Tris buffer pH 8.0 at 40 °C, rotated 15 rpm (Hybaid mini hybridization oven MK II) for up to 72 h. The reactions were then vacuum-filtered using 96-well filter plates (0.22-μm PVDF membrane) (Millipore, USA) in a Tecan liquid handler (500 mbar) (Tecan Trading AG, Switzerland). Enzymatic products in the flow-through were confirmed and quantified by mass spectrometry as well as by HPAEC-PAD and HPLC-RI/UV analyses; whereas, different concentrations (up to 1%, w/v) of the resulting xylan samples were incubated in MQ water with Novozymes xylanase NS51024 (8 × 10^−4^%, w/v) for 20 min at 40 °C at 700 rpm in a thermomixer (Eppendorf, USA). The amount of reducing sugars was quantified by the PAHBAH method [31] while the release of xylose and xylo-oligosaccharides was quantified by HPAEC-PAD analysis after vacuum filtration.

The kinetics of GOOX-Y300A (16 nM) on MeGlcA and GlcA were measured using up to 50 mM MeGlcA and GlcA in 0.3 M Tris buffer pH 8.0 at 40 °C. The amount of methyl glucaric acid was determined by measuring the release of H_2_O_2_ using a previously published colorimetric assay [23].

### Quantification of MeGlcA from glucuronoxylan

MeGlcA present in glucuronoxylan was released by a modified acidic methanolysis [32]. Briefly, glucuronoxylan (10 mg), as well as MeGlcA (1 mM), was treated with 1 mL of 2 M HCl in anhydrous methanol in glass vials at 100 °C for 3 h. Samples were then dried by nitrogen flow, and re-dissolved in MilliQ water for HPAEC-PAD analysis.

### Docking analysis

UCSF Chimera v 1.14 (https://www.cgl.ucsf.edu/chimera/) was used to energy-minimize the structures of MeGlcA (PubChem CID: 446874), glucose (PubChem CID: 5793) and GOOX wild-type (PDB ID: 2axr), as well as the structural model of GOOX-Y300A. The ligand and proteins were assigned AMBER ff14SB force field and AM1-BCC charges. Docking simulation was conducted using Autodock Vina v1.1.2 (http://vina.scripps.edu). Figures were generated by UCSF Chimera v 1.14.

### H_2_O_2_ inhibition assay

AxyAgu115A (10 μg/mL) was incubated with 1% glucuronoxylan in 50 mM Tris buffer pH 8.0 in the presence of various H_2_O_2_ concentrations (0.01–100 mM). MeGlcA (1 mM) was also incubated with the same H_2_O_2_ concentrations. The reactions were kept in the dark at 40 °C at 500 rpm for 16 h in a thermomixer (Eppendorf, USA), and then filtered using 30 kDa-cut-off filter centrifugal tubes. Catalase (200 μg/mL) was then added to the flow-through for 30-min incubation to remove H_2_O_2_ before HPAEC-PAD analysis.

### Anion-exchange chromatography

Anion-exchange chromatography was performed using Dowex 1 × 8 anion-exchange resin (50–100 mesh) in a glass column (2.6 cm ID × 30 cm) connected to a BioLogic DuoFlow FPLC unit with a Quadtec UV detector (Bio-Rad, USA) with flow rates ranging from 1 to 3.0 mL/min. Milli-Q water was used as the primary eluent, and acidic sugars were eluted using a 0–2 M ammonium acetate pH 6.5 gradient. Fractions containing eluted products were desalted and concentrated by lyophilization. The presence of sugar products in fractions was detected by spotting the sample on silica plates with aluminum backing (Sigma-Aldrich, USA), and visualized using the diphenylamineaniline stain [33].

### HPAEC-PAD and HPLC-RI/UV analyses

Reaction samples were vacuum-filtered using 0.22-μm, PVDF filter plates (Millipore, USA) with a Tecan liquid handler (500 mbar) (Tecan Trading AG, Switzerland). The flow-through was collected to Nunc™ 96-well polypropylene microplates (Thermo Fisher Scientific, USA), and covered with Nunc™ 96-well silicone cap mats. The presence of neutral and acidic sugars was detected using an ICS5000 HPAEC-PAD system (Dionex, USA) with a CarboPac PA1 (2 × 250 mm) analytical column (Dionex, USA). The HPAEC-PAD samples were eluted at 0.25 mL/min using NaOAc gradient (0–0.5 M) in 0.1 M NaOH. The same filtered samples were also analyzed by a Dionex Ultimate 3000 HPLC (Dionex, USA) equipped with a UV detector (DAD-3000) and a Shodex RI-101 differential refractive index detector. The products were separated using a Bio-Rad HPX-87H column at 65 °C, and eluted with 5 mM H_2_SO_4_ at 0.4 mL/min. Wavelengths were set at 210, 214, 260 and 280 nm. Chromatograms were analyzed using Chromeleon 7.2 (Dionex, USA).

### Nanospray ionization ion-trap mass spectrometry (NSI-MS) and LC–MS analyses

Reaction solutions were vacuum-filtered using 0.22-μm, PVDF filter plates (Millipore, USA), and then prepared in 50% methanol before being injected to a nano-ESI source on a Q-Exactive mass spectrometer (Thermo Scientific, USA) with a disposable pico-emitter. Samples were analyzed in a negative mode at a spray voltage of 2.5 kV, capillary temperature of 250 **°**C, automatic gain control target of 1 × 10^6^, injection time of 100 ms, and resolution of 140,000. Spectra were analyzed using Qual Browser in Thermo Xcalibur (v2.2) software (Thermo Scientific, USA). For LC–MS analysis, the filtered samples were analyzed using a Q-Exactive mass spectrometer (Thermo Scientific, USA), equipped with an Ultimate 3000 HPLC system (Thermo Scientific, USA) and a Hypersil GOLD column (50 × 2.1 mm) (Thermo Scientific, USA).

## Supplementary information

**Additional file 1.** Additional figures.

## Data Availability

All data generated or analyzed during the study are included in this published article.

## Abbreviations

AxyAgu115A: GH115 α-glucuronidase from *Amphibacillus xylanus*
GlcA: d-Glucuronic acid
GOOX: Gluco-oligosaccharide oxidase from *Sarocladium strictum*
MeGlcA: 4-*O*-Methyl d-glucuronic acid
