# Supplementary material for: Enzymatic production of 4-O-methyl d-glucaric acid from hardwood xylan
Source: Biotechnol Biofuels. 2020 Mar 13;13:51. doi: 10.1186/s13068-020-01691-2 (PMC7071571; doi:10.1186/s13068-020-01691-2)
Supplement: Supplementary file 1 — Additional file 1. Additional figures. [file 13068_2020_1691_MOESM1_ESM.docx]

**Enzymatic production of 4-*O*-methyl D-glucaric acid from hardwood xylan**

Thu V. Vuong^1^ and Emma R. Master^1,2*^

^1^Department of Chemical Engineering and Applied Chemistry, University of Toronto, Toronto, ON, Canada

^2^Department of Bioproducts and Biosystems, Aalto University; FI-00076 Aalto, Kemistintie 1, Espoo, Finland

**Figure S1.** SDS-PAGE of purified AxyAgu115A and GOOX-Y300A. Lane 1: Purified AxyAgu115A (2 μg, the theoretical molecular mass = 110 kDa); lanes 2 and 3: Different amounts of purified GOOX-Y300A (1 and 4 μg, the theoretical molecular mass = 56 kDa including a FAD cofactor).


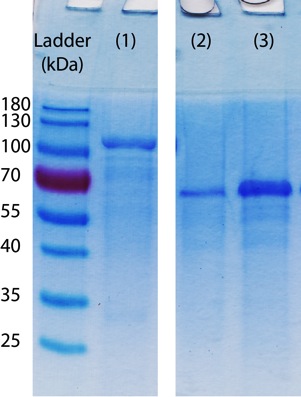


**Figure S2. HPAEC-PAD analysis of AxyAgu115A action on glucuronoxylan.** The presence of 4-*O*-methyl D-glucuronic acid (MeGlcA) was detected in a 4-h treatment of glucuronoxylan with AxyAgu115A (red line), not in the untreated glucuronoxylan sample (black line). MeGlcA at 0.25 mM (grey, dash line) was included as the standard.

**
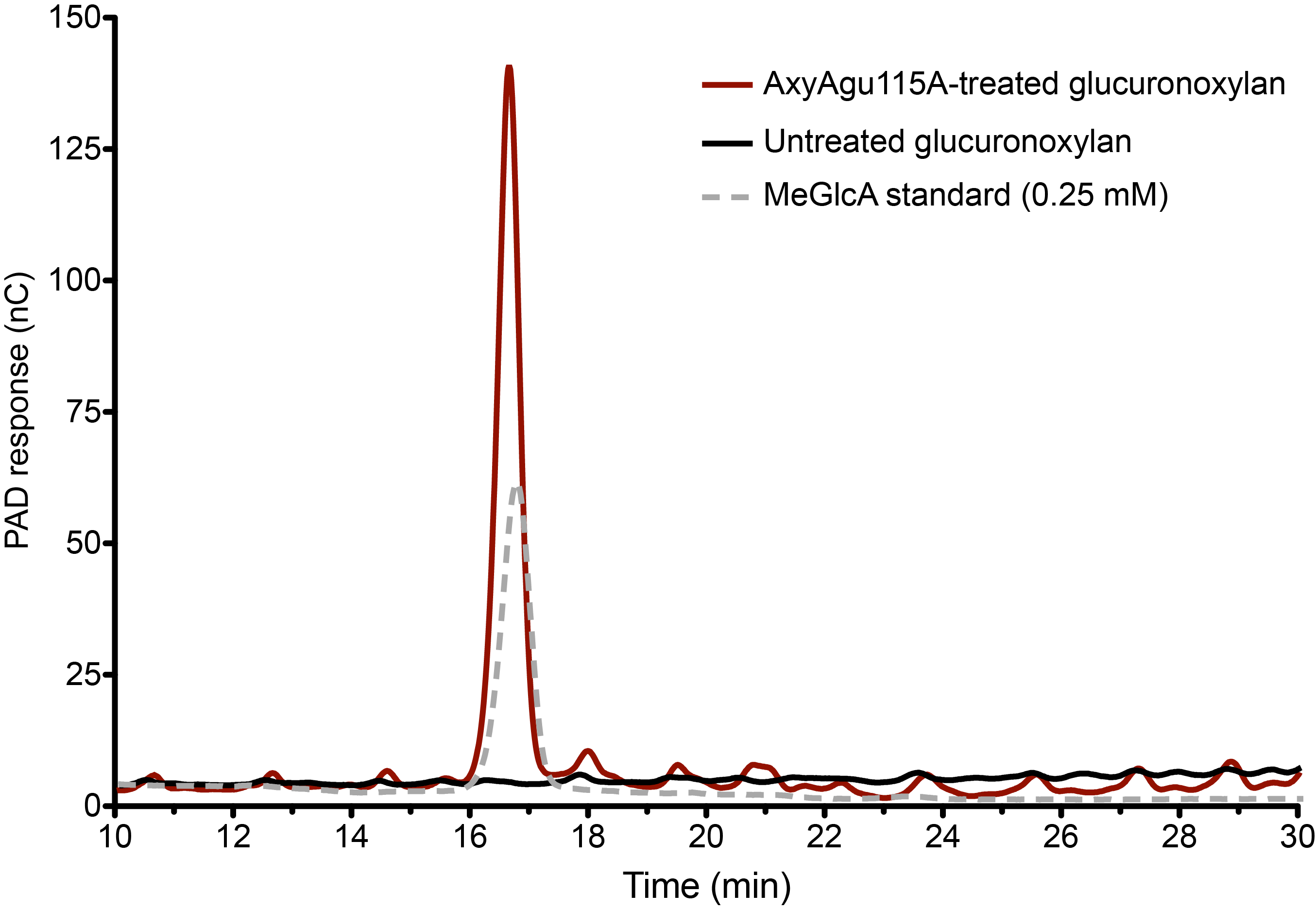
**

**Figure S3**. HPAEC-PAD chromatograms of 0.25 mM GlcA and 0.25 mM MeGlcA.


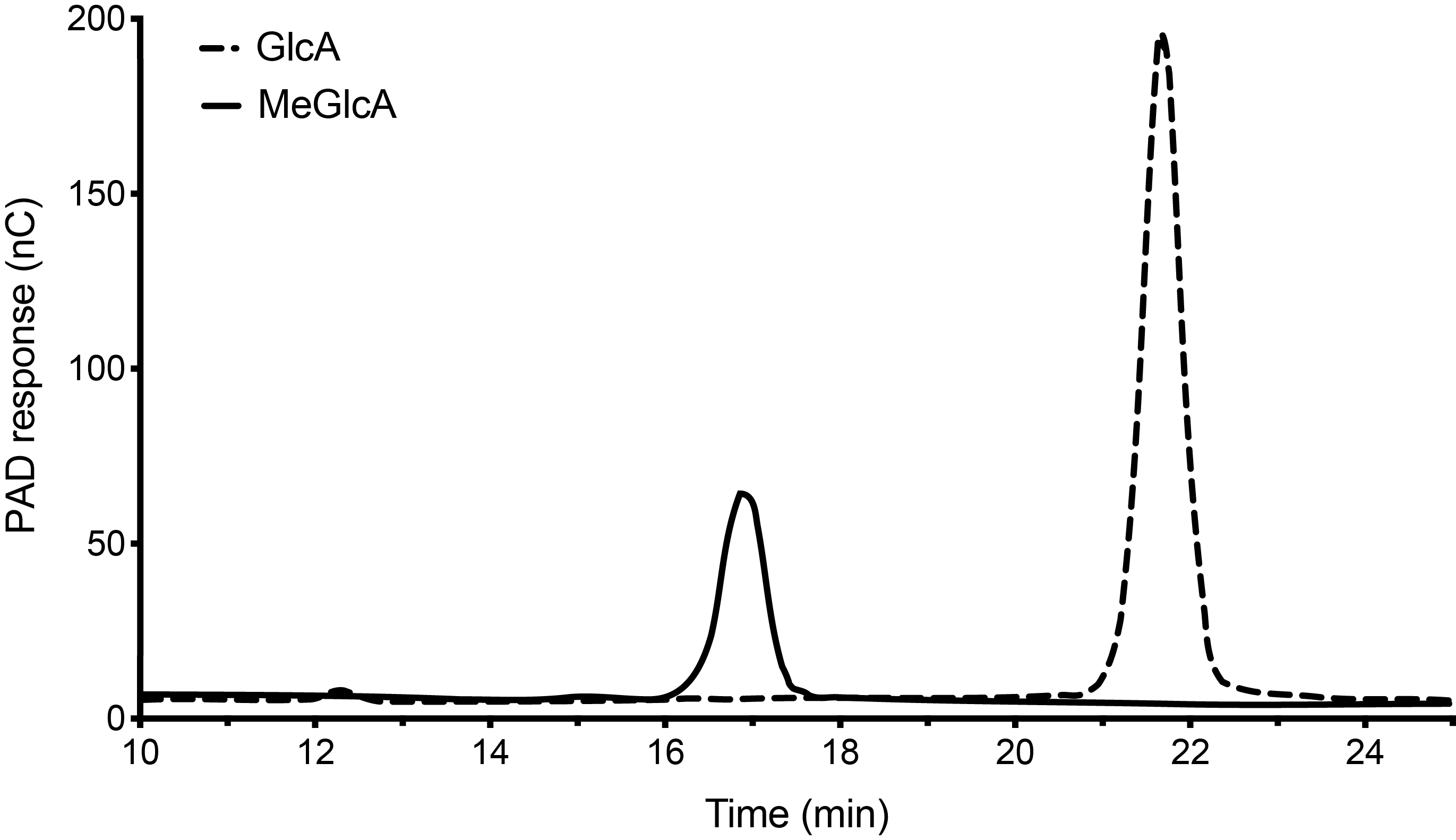


**Figure S4**. NSI-MS spectrum of released MeGlcA (208.05 g/mol) by AxyAgu115A. Samples in 50 % methanol were injected in a negative mode and the spectrum was recorded from 100 m/z to 1,000 m/z.

_
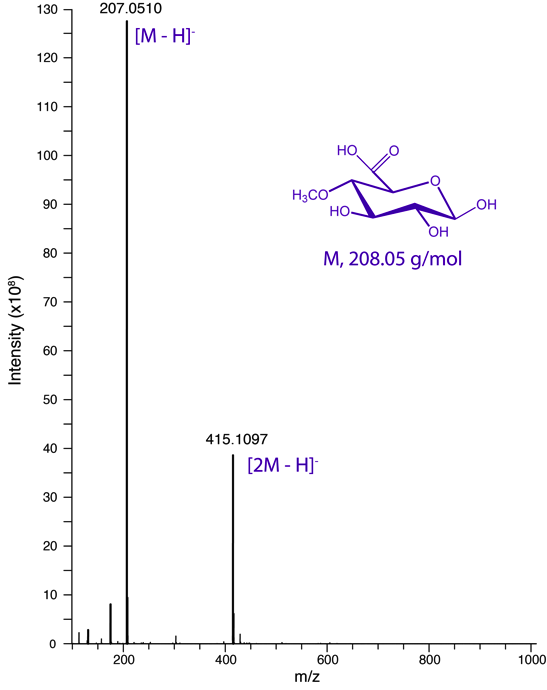
_­­

**Figure S5**. NSI-MS spectra of MeGlcA (208.05 g/mol) from experimental acquisition (A) and from simulation (B).

**
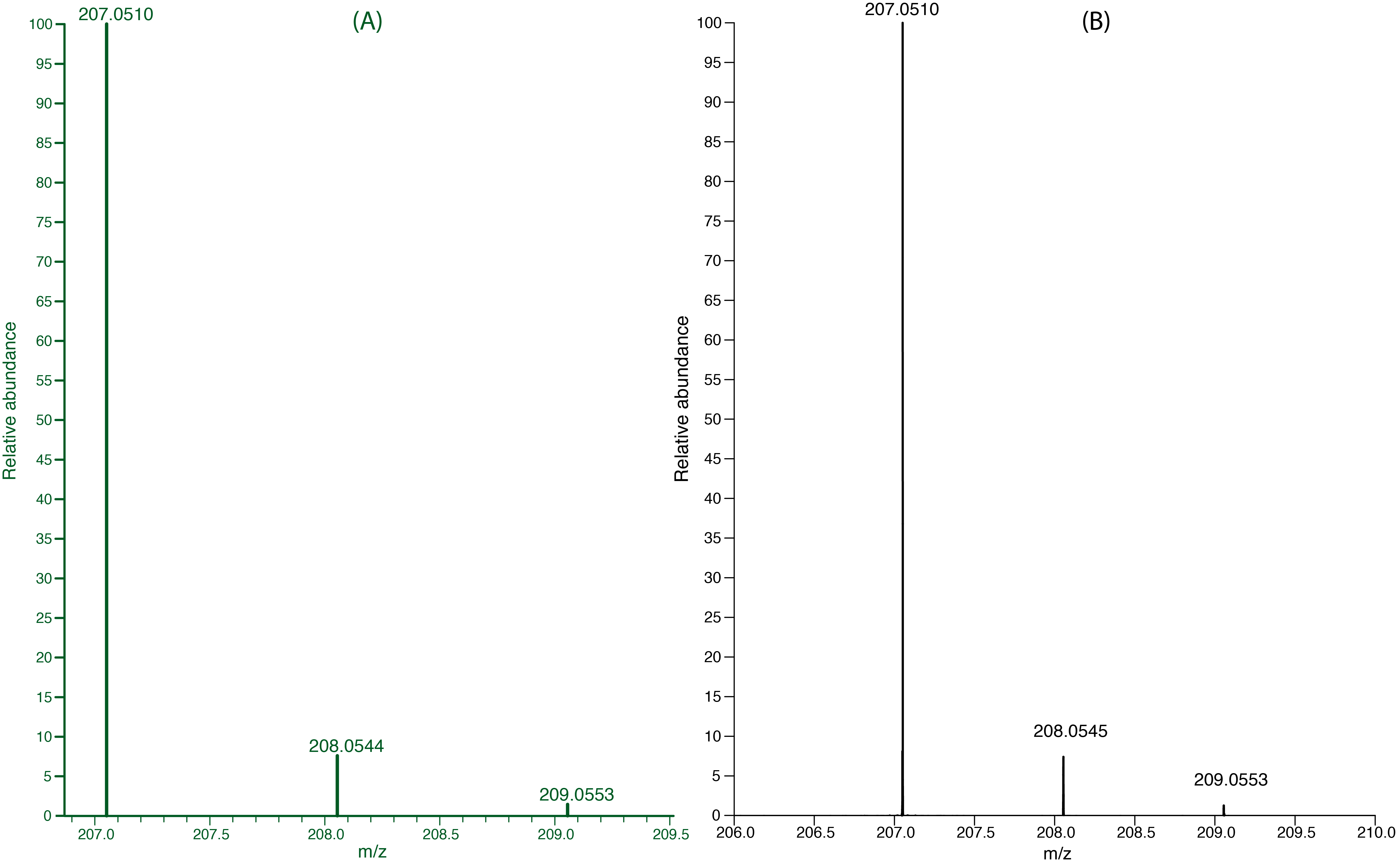
**

**Figure S6**. MeGlcA (0.05 mM - 1 mM) standard curve by HPAEC-PAD.


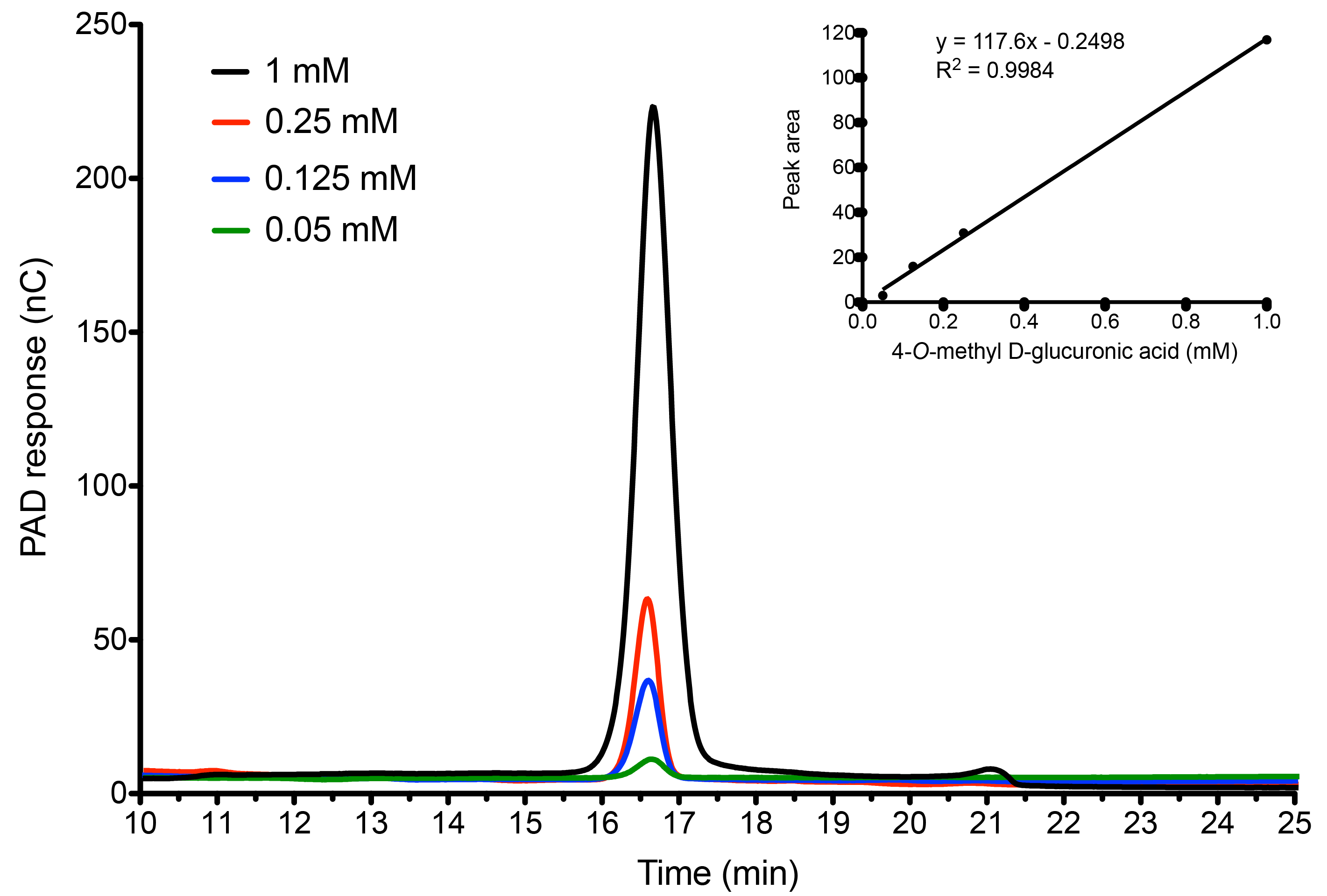


**Figure S7**. Colorimetric analysis of anion-exchange fractions resulting from AxyAgu115A digestion of glucuronoxylan. Each fraction (4 μL) was loaded on one square, numbered from 1 to 72. The silica plate was then stained with diphenylamineaniline to detect the presence of MeGlcA, which appeared when the concentration of ammonium acetate was higher than 0.5 M (from fraction 63).

**
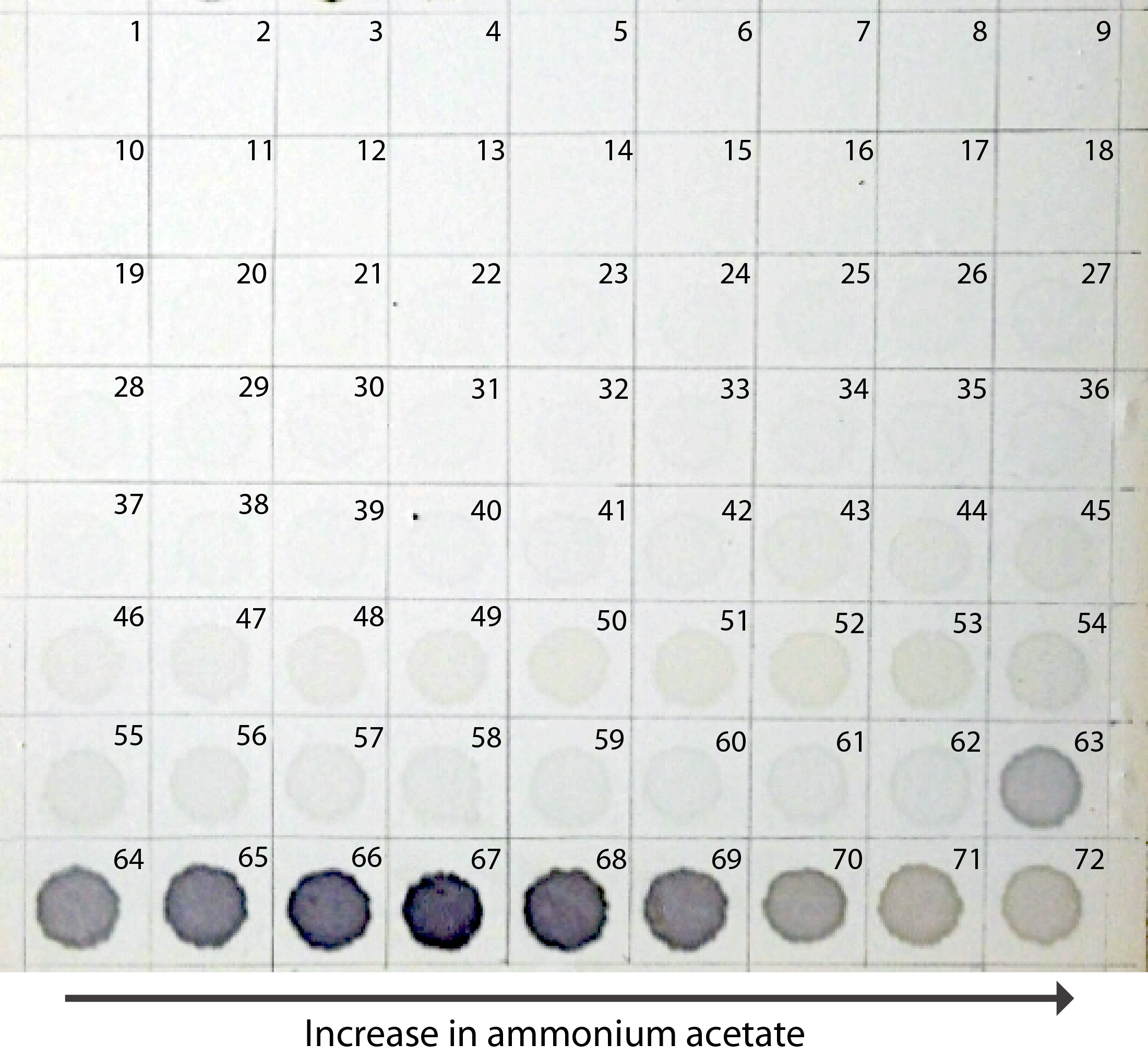
**

**Figure S8**. Activity screening of GOOX mutants and glucose oxidase (GO, cat. no. G2133 from Sigma) on GlcA and MeGlcA. The enzymes (16 nM) were assayed at 37 ^o^C with 10 mM GlcA and 1 mM MeGlcA in 100 mM Tris buffer pH 8.0 (for GOOX mutants) or 50 mM sodium acetate pH 5.0 (for GO).


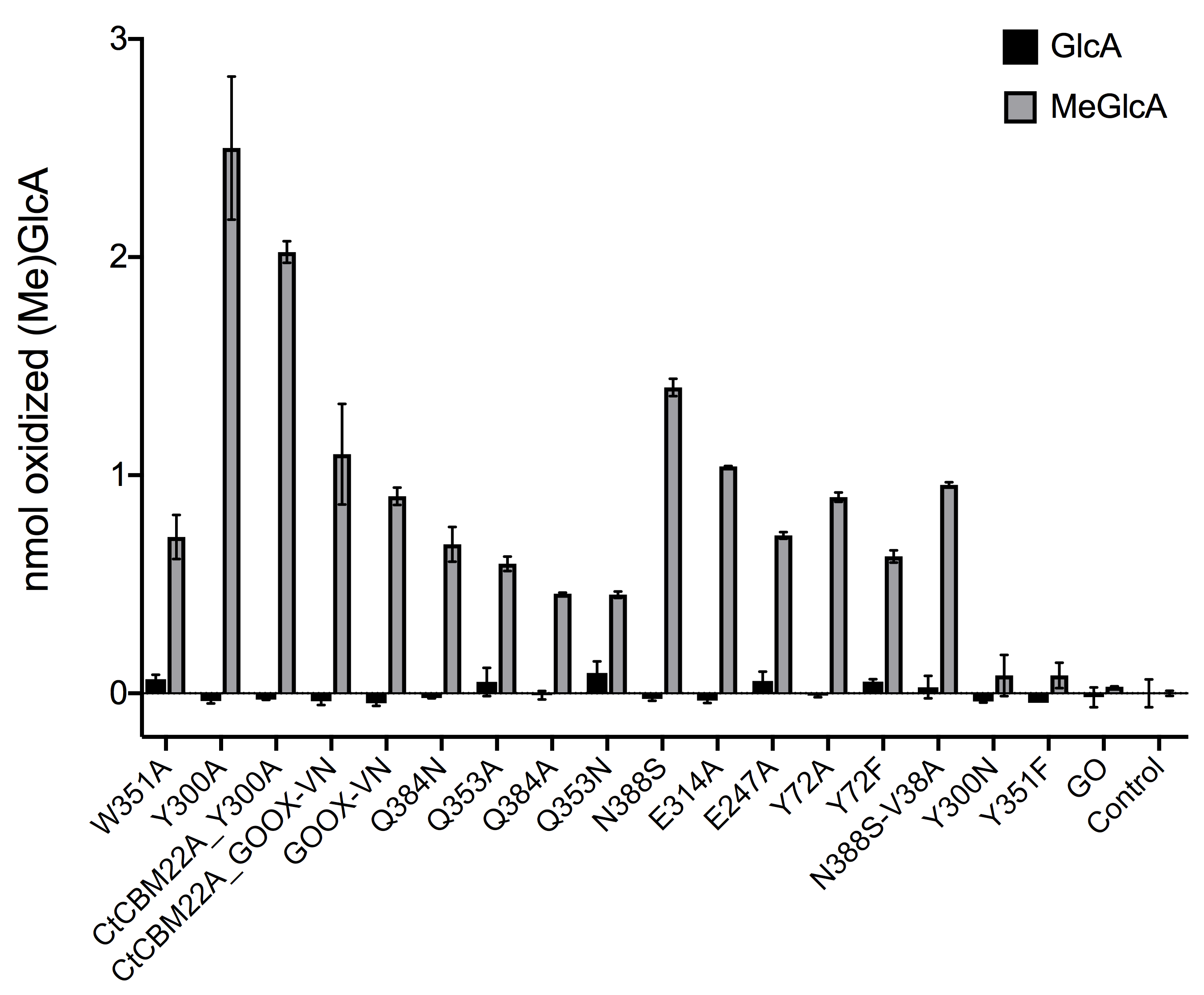


**Figure S9**. HPAEC-PAD analyses of H_2_O_2_ effects on AxyAgu115A activity and MeGlcA degradation. (A) The presence of H_2_O_2_ did not cause a loss of MeGlcA (as quantified by peak area). (B) Higher concentrations of H_2_O_2_ lowered the amount of MeGlcA released from glucuronoxylan by AxyAgu115A.


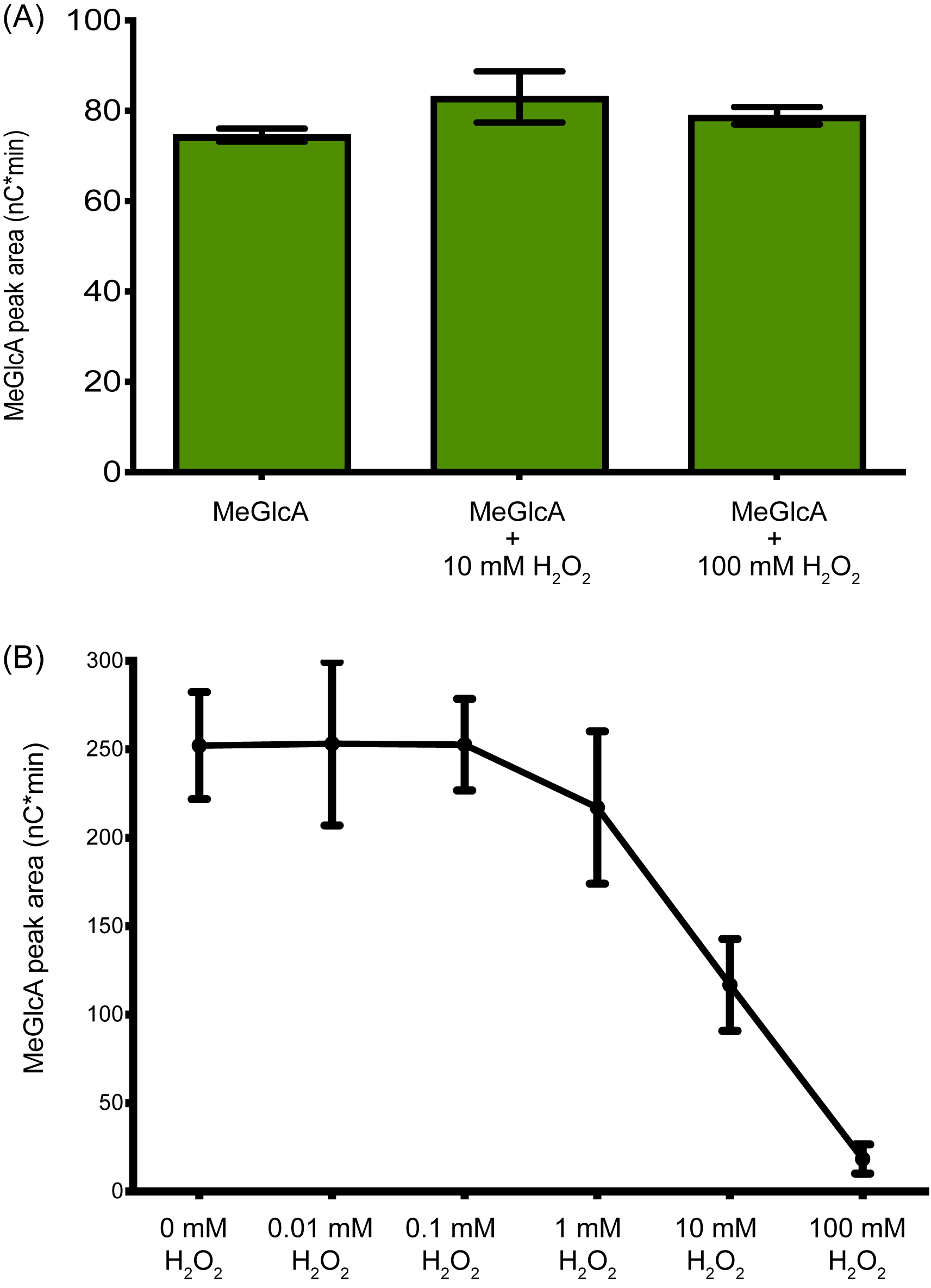


**Figure S10. Isolation of xylan after AxyAgu115A and GOOX-Y300A treatment.** Untreated glucuronoxylan remained soluble before (A) and after (C) centrifugation; however, hydrogel-like material was formed (B) in the reaction incubated with the two enzymes, and it was separated out by centrifugation (D).

**
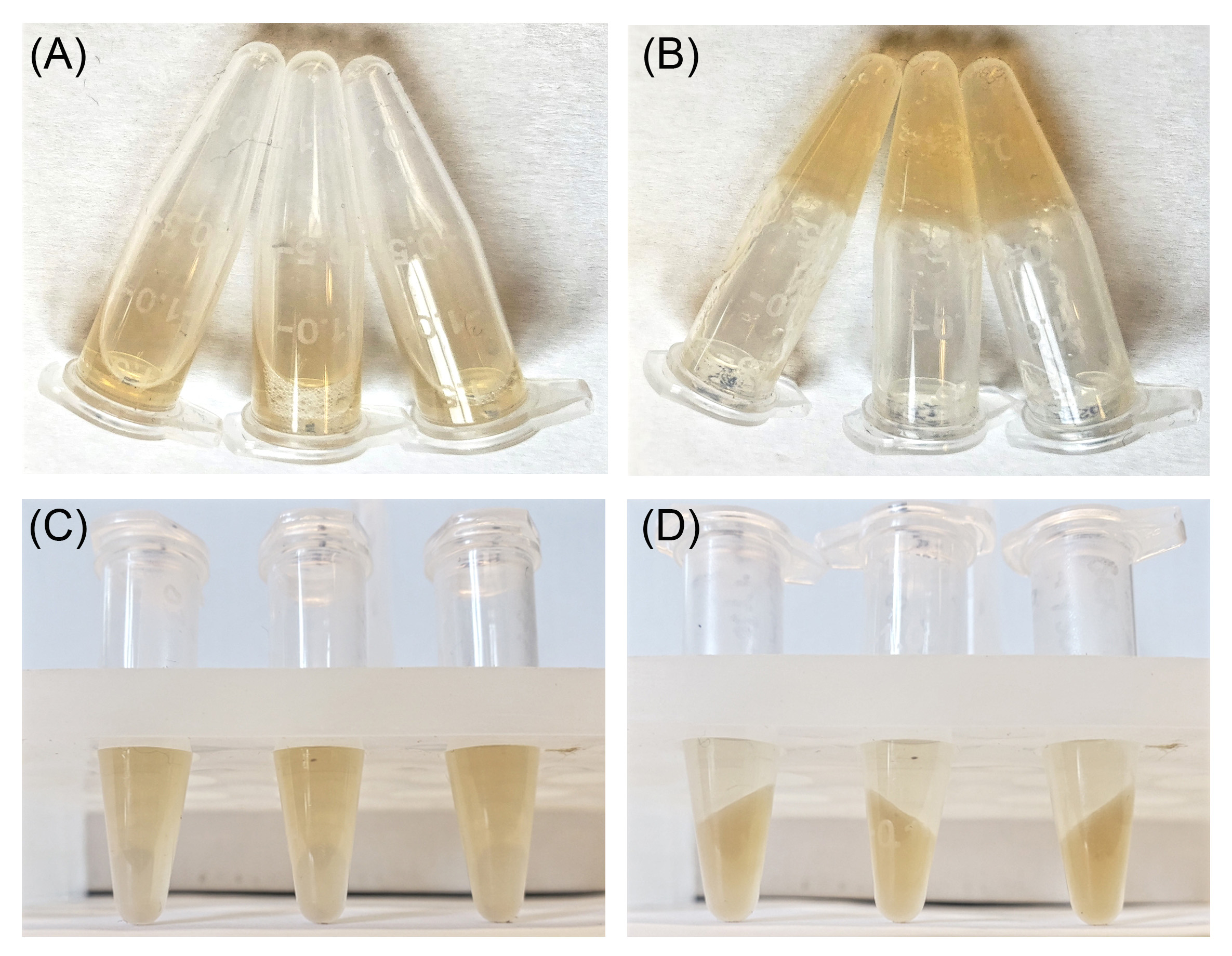
**

**Figure S11**. Reducing-end sugars released from glucuronoxylans before (triangle) and after (circle) AxyAgu115A and GOOX pre-treatments. After the two-enzyme incubation, the xylan fraction was harvested and water-washed by centrifugation before xylanase digestion. The amount of reducing-end sugars was measured by the PAHBAH method.
